# Accurate population estimation of *Caprinae* using camera traps and distance sampling

**DOI:** 10.1038/s41598-020-73893-5

**Published:** 2020-10-20

**Authors:** Grant M. Harris, Matthew J. Butler, David R. Stewart, Eric M. Rominger, Caitlin Q. Ruhl

**Affiliations:** 1 United States Fish and Wildlife Service, Albuquerque, NM USA; 2 New Mexico Department of Game and Fish, Santa Fe, NM USA

**Keywords:** Population dynamics, Ecology, Conservation biology

## Abstract

With most of the world’s *Caprinae* taxa threatened with extinction, the IUCN appeals to the development of simple and affordable sampling methods that will produce credible abundance and distribution data for helping conserve these species inhabiting remote areas. Traditional sampling approaches, like aerial sampling or mark-capture-recapture, can generate bias by failing to meet sampling assumptions, or by incurring too much cost and logistical burden for most projects to address them. Therefore, we met the IUCN’s challenge by testing a sampling technique that leverages imagery from camera traps with conventional distance sampling, validating its operability in mountainous topography by comparing results to known abundances. Our project occurred within a captive facility housing a wild population of desert bighorn sheep (*Ovis canadensis*) in the Chihuahuan desert of New Mexico, which is censused yearly. True abundance was always within our 90% confidence bounds, and the mean abundance estimates were within 4.9 individuals (average) of the census values. By demonstrating the veracity of this straightforward and inexpensive sampling method, we provide confidence in its operability, urging its use to fill conservation voids for *Caprinae* and other data-deficient species inhabiting rugged or heavily vegetated terrain.

## Introduction

Ecologists seek accurate abundance estimates for evaluating animals threatened status, targeting conservation actions, building ecological relationships, managing game and establishing harvest regimes. When species, such as *Caprinae*, inhabit topographically complex, heavily vegetated and remote terrain, their task grows complicated by methodological, logistical and economic constraints. Here’s the rub: Abundance estimators applied to unmarked animals often generate bias by failing to meet sampling assumptions when densely vegetated and rugged terrain limit animal observation and detection, costs to properly implement the technique exceed project budgets, or the technique contains untenable logistics (i.e. aerial surveying within no flight zones). It is unsurprising then, that of the world’s 103 *Caprinae,* the International Union for Conservation of Nature (IUCN) considers over 70% threatened, with most lacking abundance and distribution data, thereby compounding their dire conservation status^1^. Similarly, other endangered mammals, like the recently discovered silver-backed chevrotain (*Tragulus versicolor*) and the secretive fishing cat (*Prionailurus viverrinus*) lack practical and effective techniques for acquiring abundance data, constraining conservation work^2,3^.

We’re motivated to design a technique producing abundance and distribution data for *Caprinae* and other mammals inhabiting remote or highly vegetated terrain. In these situations, traditional sampling approaches can be ill-suited or unaffordable. Aerial sampling techniques like double-observer account for detecting animals when visible, but not their availability to be observed. When animals conceal themselves (e.g. in ravines, caves or vegetation) they become unavailable for detection, biasing abundance estimators low^4^. Further, aerial flights are often cost prohibitive or untenable for *Caprinae* inhabiting countries imposing no-flight zones^1^. Other techniques of population estimation, like mark-capture-recapture and sightability modeling account for animal detectability and availability by marking animals, typically with telemetry (which also supplies mortality and movement data). Capturing and marking animals increases project costs (e.g. equipment, helicopter time for captures), notwithstanding the requirement of developing correction functions unique to each survey situation^4–6^. Grid-based, DNA assays produce accurate abundance estimates, provided all individuals have an equal probability of capture. This assumption is often violated and the work is costly^7^. Some biological programs default to minimum counts by reducing flight time, or using ground-based crews. Minimum counts identify harvestable animals and ratios between adult females and young. The technique does not produce measures of precision and the exactness of the count is usually unknown (except in rare situations like captive settings), frustrating their ability to relate trends with ecological factors or monitor the efficacy of conservation and management actions. Indeed, challenges with the affordability and proper implementation of these traditional monitoring efforts, plus the limitations in interpreting simple counts to monitor population trends, could lead to erroneous conclusions about population status^4,7^.

To address these cost and operational challenges, recent studies employ camera traps to estimate species abundances in the absence of marked animals^8–11^. These approaches, however, need additional information (e.g. movement rates, home range sizes) which may be unavailable, or the methods remain theoretical and untested in real world situations (i.e. random encounter), highlighting the need for alternatives. Such work also requires validation for ensuring they produce credible abundance estimates. Validation also builds confidence in the approach and its application to inform real-world conservation problems.

**Figure 1:**
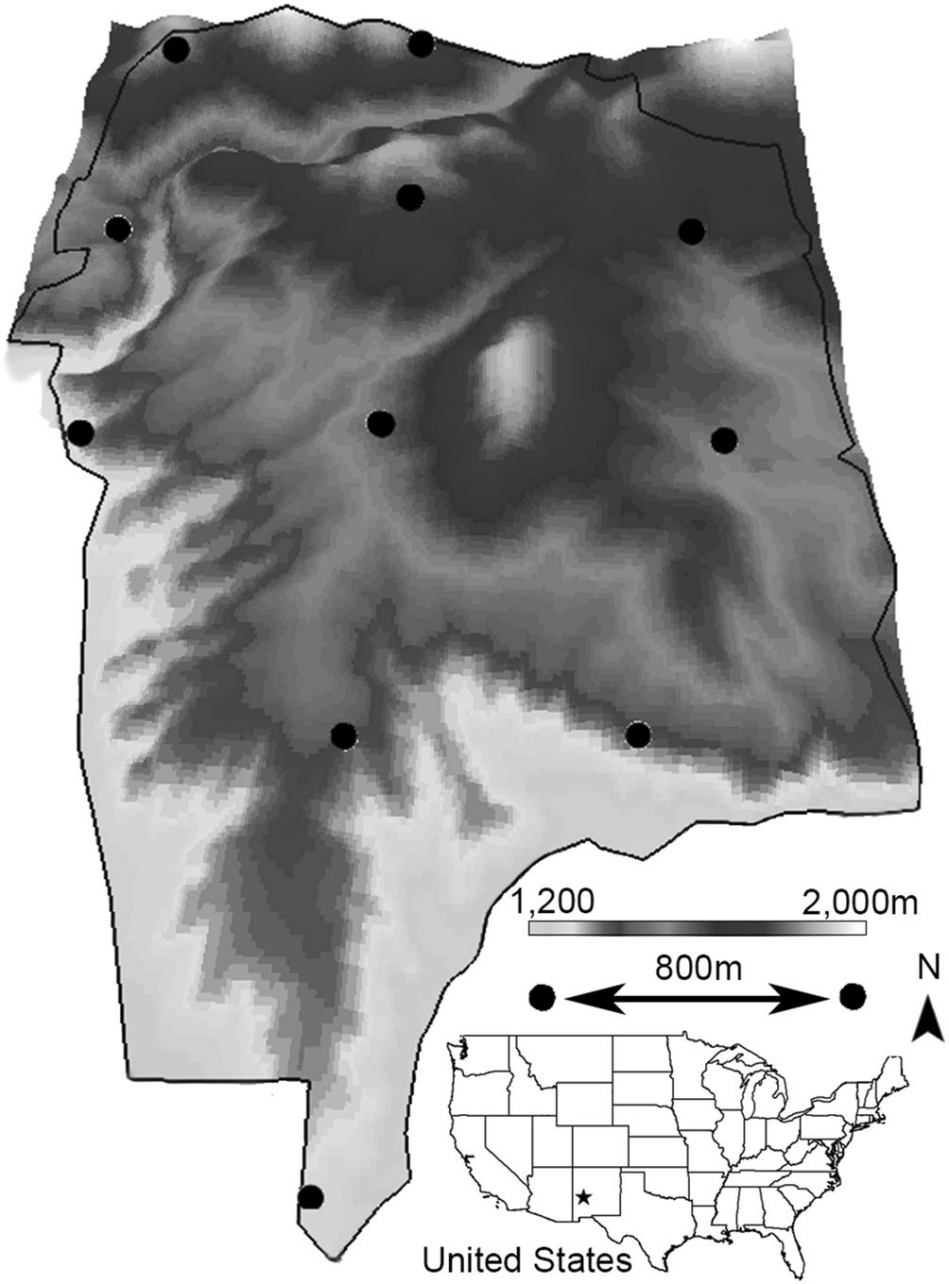
Topographical map of the Red Rock facility which contains a population of desert bighorn sheep near Red Rock, New Mexico (area = 6.2 km^2^; elevation range 1200–2000 m; black star identifies the location within the United States subset). Within this penned facility (black border), the New Mexico Department of Game and Fish censuses sheep numbers annually. We used imagery gained from 11 trail cameras spaced along an 800-m grid with a random geographical start (black circles; the three dimensionality distorts distance perception), and distance sampling approaches to estimate the abundances of desert bighorn sheep within this facility. Comparisons between our abundance estimates and truth (gained from the censuses) demonstrate the veracity of this estimation technique (map generated with ArcGIS Desktop 10.6 software; https://www.esri.com).

We build off these techniques using distance sampling with imagery from camera traps to estimate animal abundance^10^, and validate the approach by comparing results to known abundances. Our project occurred within a 6.2 km^2^ captive facility housing wild desert bighorn sheep (*Ovis canadensis*) in the Chihuahuan desert of New Mexico (Fig. 1). The New Mexico Department of Game and Fish (NMDGF) annually censuses this population, providing a unique situation to test the veracity of the camera trap-based distance sampling technique.

## Results

**Figure 2:**
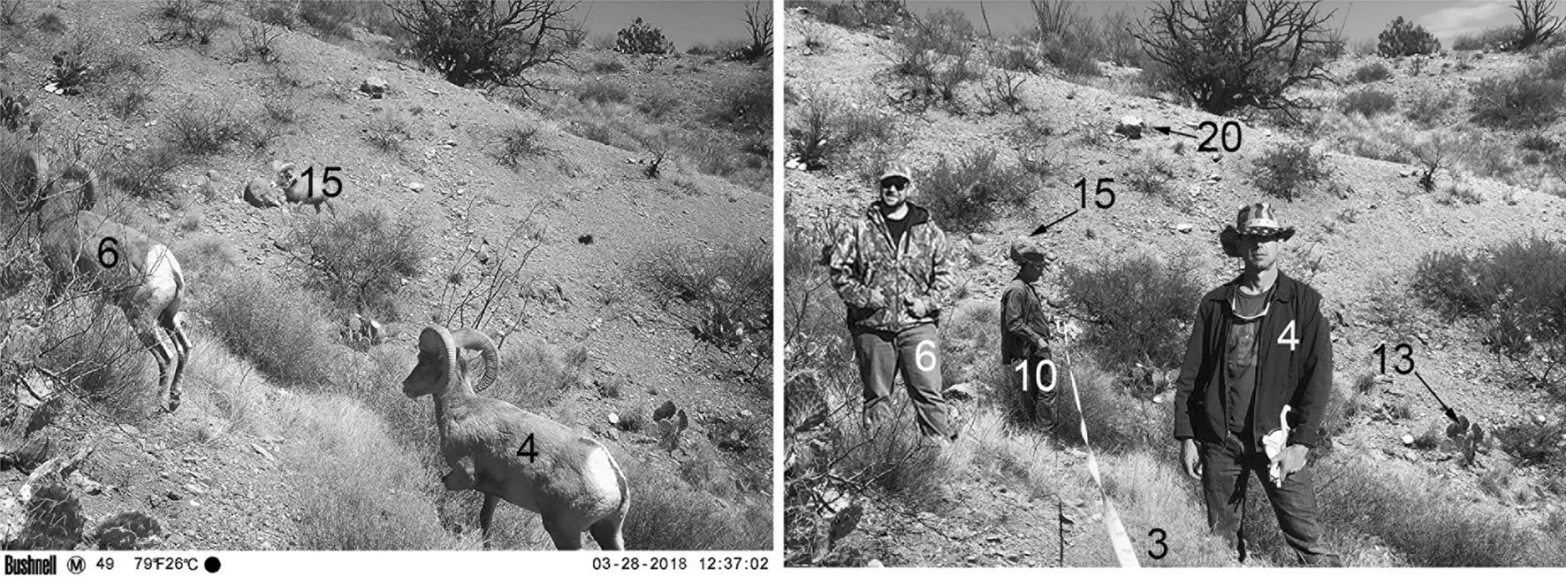
We measured the distances between desert bighorn sheep and the trail cameras imaging them, by printing out all of the sheep images, visiting the site, and positioning a human in the exact location that the sheep was imaged. We then used a range finder or metric tape to measure the distances. We supplemented these measurements by recording distances between the camera and several recognizable objects in the images (e.g. rocks, plants), to ensure accurate distance delineations for the sheep.

**Figure 3:**
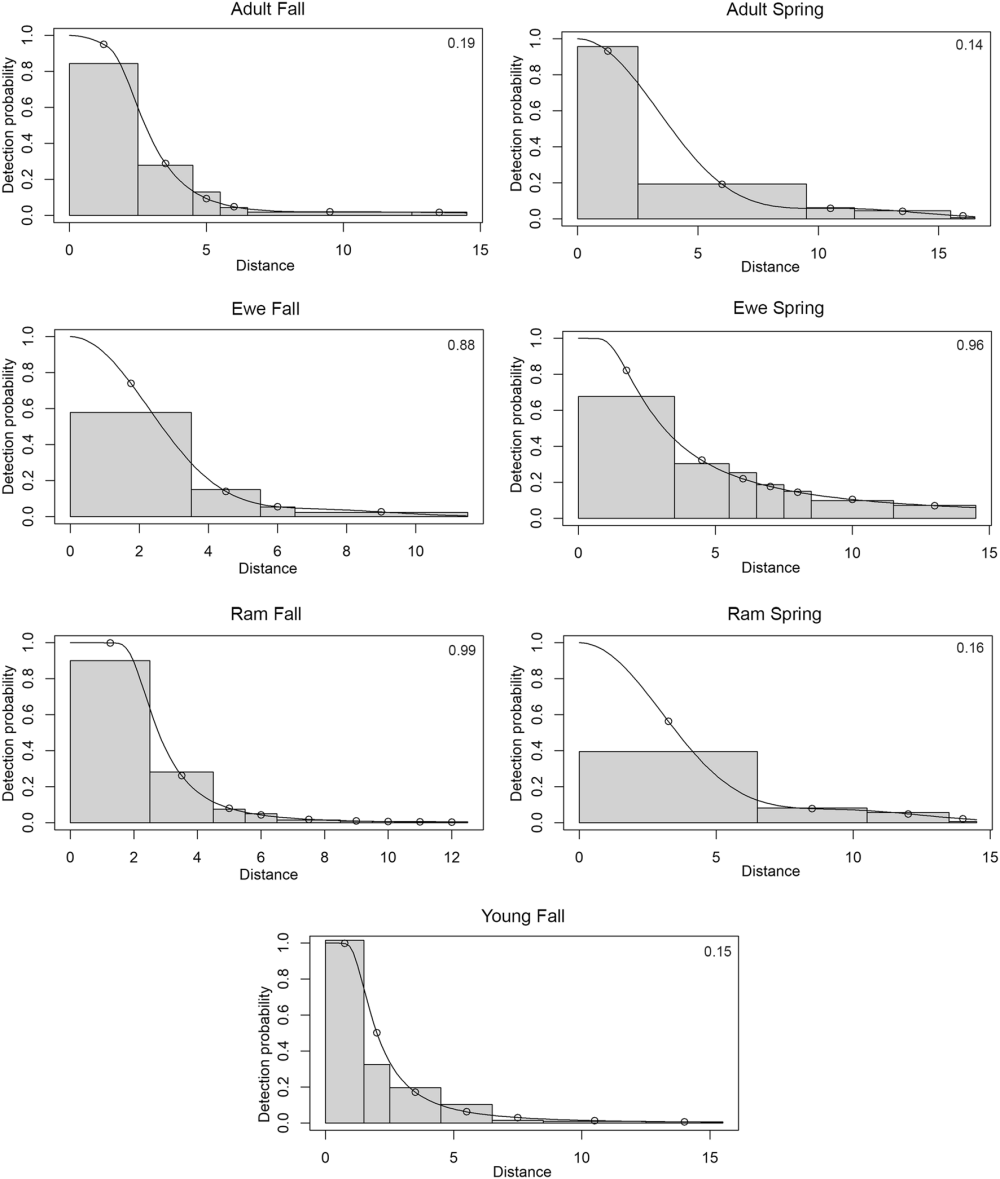
Histograms describing detection probability with the distances of desert bighorn sheep to trail cameras that imaged them at the Red Rock facility, for each period, age and sex category. Solid lines indicate the fitted detection function from the distance models receiving most AIC support (Table S1). Model fit was evaluated by the goodness of fit test (GOF), which indicated models fit well [p-values; upper right value in each plot; compare predicted (open circles) with mean observed for each bin (histogram)]. Only the model receiving most AIC support is pictured for ewes during the fall and spring study periods.

Between 1 October 2017 and 1 May 2018 we collected 3,122 total images from 11 camera traps spaced along an 800-m grid within the pen (Fig. 1). Desert bighorn sheep constituted 559 total images. The majority of other mammal species imaged included white-nosed coati (*Nasua narica*), Coues deer *(Odocoileus virginianus couesi*), collared peccary (*Pecari tajacu*) and mountain lion (*Puma concolor*). We used distance measures between the camera and photographed desert bighorn sheep (mean distances = 5.4 m; SD = 5.6 m; range = 0–40 m) to estimate abundances with the program R ‘distance’ package (version 0.9.8; Fig. 2^12^). We analyzed data using a fall/early winter period (hereafter termed fall: 1 October 2017–31 January 2018) and a spring period (1 March 2018–1 May 2018). We selected models based on AIC criteria, and all models receiving most AIC support passed goodness of fit tests (GOF; Fig. 3). All of our models had robust fits, and we based abundance estimation from them with confidence (Fig. 3; Supplementary Fig. S2).

The Red Rock facility contained 53 adult desert bighorn sheep (animals > 1.5 years) in May 2017 and 69 adults in May 2018. We estimated 54.9 adult sheep (90% CI 24.6–122.6; CV = 0.47) for the fall period and 66.0 (90% CI 32.2–135.3; CV = 0.42) during the spring period (Table 1; Supplementary Table S1).

**Table 1:** Seasonal census results (*N*), abundance estimates ($\widehat{N}$), 90% confidence limits (LCL: lower; UCL: upper) and coefficient of variation (CV) for desert bighorn sheep inhabiting the Red Rock facility within the Chihuahuan desert of New Mexico, USA.

Category^a^	5/15/2017 Census *N*	Fall estimates				5/1/2018 Census *N*		Spring estimates				
		LCL	$\widehat{N}$	UCL	CV			LCL		$\widehat{N}$	UCL	CV
Rams	25	6.45	21.41	71.11	0.75	30		10.29		27.37	72.79	0.59
Ewes^b^	28	10.32	24.28	57.54	0.53	39		6.76		24.24	87.72	0.70
Adults	53	24.59	54.91	122.61	0.47	69		32.15		65.96	135.30	0.42

^a^The categories rams, ewes and adult (rams + ewes) includes animals > 1.5 years old.

^b^Abundance estimates represent model averages for ewes.

The pen contained 25 rams and 28 ewes during May 2017 and 30 rams with 39 ewes in May 2018. We estimated 21.4 rams (90% CI 6.4–71.1; CV = 0.75) and 24.3 ewes (90% CI 10.3–57.5; CV = 0.53) using the fall 2017 data. During the spring period of 2018, we estimated 27.4 rams (90% CI 10.3–72.8; CV = 0.59) and 24.2 ewes (90% CI 6.8–87.7; CV = 0.70; Table 1; Supplementary Table S1).

We estimated a mean of 20.0 young desert bighorn sheep (animals ≤ 1.5 years) during the fall period (90% CI 8.0–49.5; CV 0.57), with a mean young:ewe ratio of 82.2:100. Young sheep experience higher mortality than adults^13^, so by fall, an unknown number of lambs had died. Indeed, mountain lions (*Puma concolor*) entered the pen during our study, and although quickly removed, they generated an unknown amount of predation. The lack of a fall census prevents comparing our estimate of young sheep to a known value. Lambing also began just prior to the May 2018 census, hampering the estimation of young during spring.

## Discussion

Our method accurately estimated the abundance of adult desert bighorn sheep. True bighorn sheep abundance was always within our 90% confidence bounds, and our mean abundance estimates were on average within 4.9 individuals of the census values, validating that distance sampling from camera traps produces reliable results. Exact matches between estimates and census numbers cannot be expected because sampling error is natural.

Biologists often aim to estimate animal abundances with CVs ≤ 0.2^5^. Our precision was lower because desert bighorn sheep cluster, and did not distribute themselves similarly across temporal periods, thereby generating greater variability in abundance among camera trap sites. For example, we acquired a third less observations during the 4-month fall survey period than the 2-month spring survey period.

Data collection spanned multiple months to ensure sufficient numbers of detections for analyses. As study longevity increases, however, so does the likelihood of sampling an open population*.* Were longer periods necessary to accumulate sufficient amounts of data, then population estimates reflect births and deaths during the study, plus immigration and emigration in non-captive populations.

Our project relied on one image per camera trigger. Were cameras set with a burst mode, it could easily triple the workload of image sorting and identification (assuming a burst of 3 images) while potentially introducing issues of overdispersion^14^. The technology in other trail cameras enable the recording of images at defined intervals (e.g. 30 s, 60 s). Using a 60 s interval during the duration of our study would generate over 300,000 images per camera. Image loads of these magnitudes likely require leverage from artificial intelligence solutions to identify and count the number of animals in the images [such as Microsoft’s MegaDetector (https://github.com/microsoft/CameraTraps/blob/master/megadetector.md)], yet such solutions do not identify the species of animal nor do they estimate distances between the animal and the trail camera imaging them.

We inform the design of projects estimating animal abundances elsewhere, by evaluating tradeoffs between CV and number of camera trap locations (i.e. sampling sites^15^). Our study relied on 11 sampling locations and because of the high variation in encounter rates, obtaining a CV of 0.2 would require 48 sampling locations for adult desert bighorn sheep within the spring study period (2 months) and 62 cameras for the fall period (4 months; Supplementary Fig. S3). Species with 25% less variability in encounter rate with a similar detection process would require approximately half of these sampling locations to achieve a CV of 0.2. Relaxing precision to 0.3 CV would provide valuable information for conserving many of the *Caprinae* populations lacking abundance and distribution data. In this case, the number of sampling locations required would be 21 and 27 given our encounter rates for the spring and fall periods, respectively.

Since the number of observations we used to model the detection process were usually large (> 100), most of the variation in our abundance estimates (> 90%) was due to encounter rates, not detection probability. Therefore, even though *Caprinae* populations typically span 100 s of km^2^ with densities ~ 1/km^2^^1^, the variability in encounter rate, not the geographical extent, dictates sampling effort. As variation in encounter rate increases, the number of cameras required to reduce CV does too^15^. By performing simple pilot studies, practitioners can determine the variability in encounter rates to calculate the sample size requirements for meeting their precision goals. Stratified sampling (e.g., by animal density, habitat quality) would further reduce variability. Even if 100 camera traps were needed (< $15,000 U.S.) to cover a wild, expansive population, this initial investment would be a fraction of the costs required of helicopter surveys (were flights even permissible^1,4^), including technician time, with the equipment lasting multiple years.

Including covariates as predictor variables and combining age and sex categories into the same model could increase estimate precision. We chose not to pursue this step, because the procedure primarily reduces variation in the detection process, and in our case, most variation stemmed from the encounter process. We also did not expect to acquire different detection functions by category (e.g. ram, ewe), season or time of day (Fig. 3).

We demonstrate that a camera trap-based, distance sampling method offers an inexpensive, simple and accurate approach to estimate abundances of *Caprinae*. The method is accessible to practitioners and overcomes the deficiencies inherent to conventional sampling procedures attempting to estimate the abundances of animal populations. We encourage widespread implementation of this approach to fill knowledge gaps of rare *Caprinae* and other species inhabiting heavily vegetated or remote terrain to help steer their preservation and management.

## Methods

We estimated the abundance of desert bighorn sheep in a captive facility located within the Chihuahuan Desert of New Mexico (Fig. 1). The area is arid, mountainous, with steep cliffs punctuated by ravines. The entire facility is enclosed by a high fence, preventing desert bighorn sheep ingress or egress. These animals are wild, unconditioned to humans, and used by The New Mexico Game and Fish Department (NMDGF) to establish and bolster other desert bighorn sheep populations within New Mexico^16^.

NMDGF leads an annual spring census to enumerate desert bighorn sheep numbers within the facility. Methodologically, the census uses a ground crew of spaced individuals walking in a line perpendicular to pen fencing, within the facility (i.e., drive count or census). Each individual keeps track of neighboring individuals to space the line, and to count any desert bighorn sheep breaking past them. Most desert bighorn sheep are herded ahead of the line. Other biologists at high topographical sites use spotting-scopes and binoculars to count and age class the moving sheep. Census counts are classified by animal ages and sex. We consider young sheep any animal ≤ 1.5 years old. Adult rams and ewes consist of males and females aged > 1.5 years old, respectively. The group “adult” includes all sheep > 1.5 years old.

We established 11 motion activated camera traps (Bushnell Trophy Cam) at the centers of an 800-m grid with a random geographical start. Cameras were secured to T-posts or existing vegetation when rocky areas thwarted post establishment. Most cameras were oriented north, to minimize sun exposure in the imagery. When vegetation or rock blocked the camera view, the camera orientation was rotated eastward until a clear view was obtained. Camera heights were 0.9–1.2 m with declination perpendicular to the ground. Cameras were checked at 6 month intervals and the retrieved SD cards were never full. Cameras were motion activated and set at the shortest delay possible (10 s; meaning that the camera waits at least 10 s after recording a picture before it will record another). In practice, the fastest trigger speed that cameras recorded imagery was a mean of 14.92 s (N = 6 cameras; SD = 0.94), a value we rounded to 15 s and employed in our analyses. Cameras recorded one image per trigger. We deployed cameras by 15 May 2017 and retrieved cameras no earlier than 30 April 2018. Our analyses period began on 1 October 2017 and ended on 1 May 2018. We employed a 5 month acclimation period to avoid the cameras serving as a novel attractant for desert bighorn sheep, which would violate distance sampling assumptions. Further, we experienced 1 camera failure during this acclimation period, and relocated 2 misplaced cameras. Lastly, some desert bighorn sheep gathered in shady locations near cameras during hot months (June–September) which created extreme variation in the encounter rate. The 1 October 2017–1 May 2018 period lacked all of these issues.

Imagery of desert bighorn sheep were identified and then classified by sex and age class: rams, ewes, young, adults (an adult-sized animal with sex undiscernible), and unknown [undiscernible (e.g. picture of a hoof, or animals blocking a clear view another animal)^17^]. To quantify distances, an individual stood at each camera and used the printed images of desert bighorn sheep to position another person at the exact locations that an animal was imaged. Distances between individuals were measured with a laser rangefinder and metric tape (Fig. 2; the authors confirm that informed consent was obtained to publish the identifying information/images in an online open-access publication). We supplemented these measurements by recording distances between the camera and several recognizable objects in the images (e.g. rocks, plants), to ensure accurate distance delineations for the imaged sheep.

The sensitivity of a trail camera’s passive infrared sensor (PIR) will decline as radial distance from the camera increases. Other factors, like vegetation and topography, also cause animal detections to decline with distance. This situation makes distance sampling an appealing approach, as its’ foundational premise is that the probability of detecting an animal declines with distance from the observation point^15^. The technique relies on measured distances between animals and the observation point. Therefore, we used the distance measures of desert bighorn sheep to the respective trail camera imaging them, to estimate sheep abundances using the ‘distance’ package (version 0.9.8^12^) in program R. We analyzed data using a fall period (1 October 2017–31 January 2018) and a spring period (1 March 2018–1 May 2018). Dates for these periods were selected by season while ensuring sufficient sample sizes.

The camera trap operates like a point count sample. The sampling angle for each camera’s field of view was 50°^18^. Therefore, we multiplied sampling effort by this fraction (50/360) to correctly represent the amount of sampling area.

**Figure 4:**
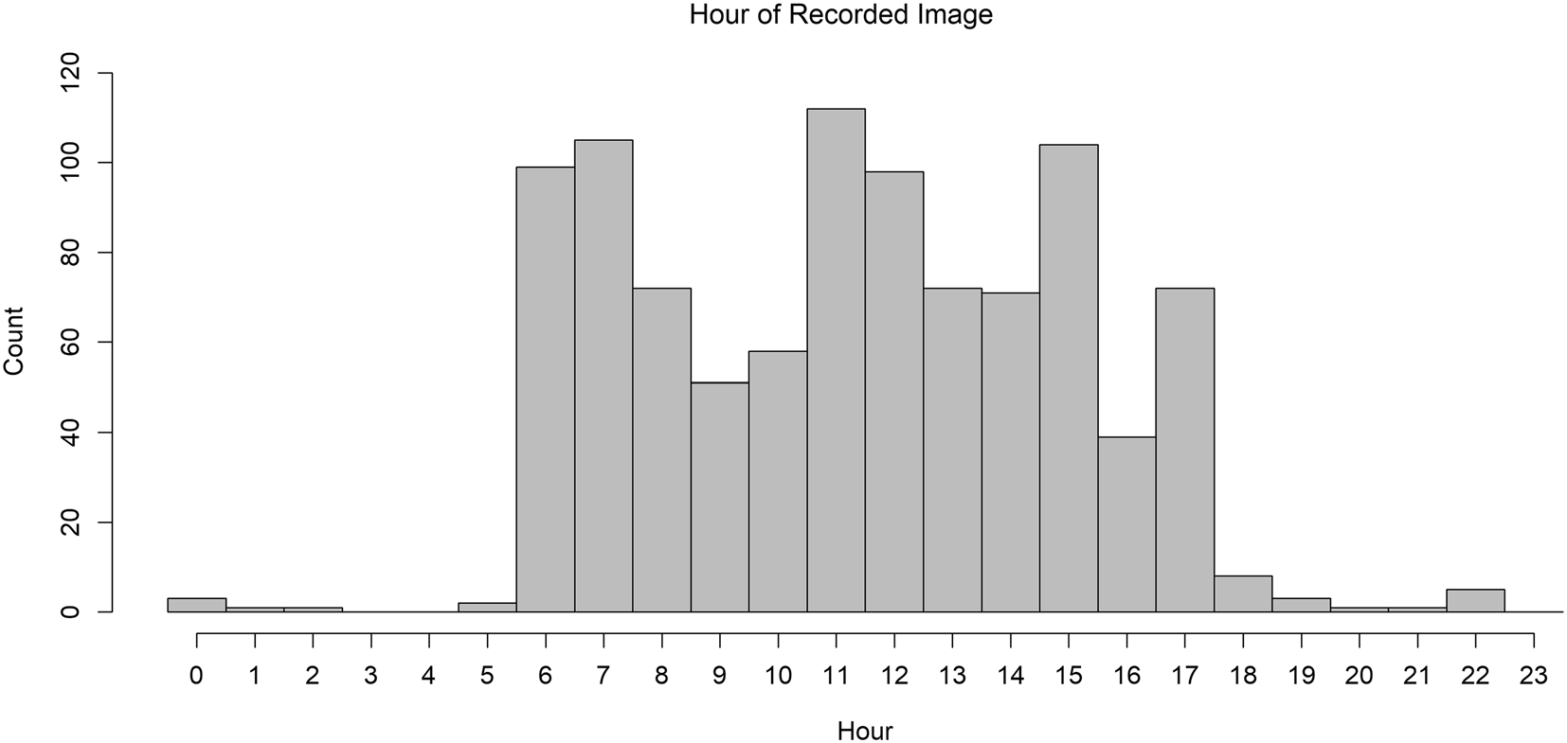
Histogram depicting the number of all desert bighorn images taken at the Red Rock facility (count) within hourly intervals during the study period (10/01/17–05/01/2018).

When analyzing data within a distance sampling approach, obtaining unbiased density estimates relies on correspondence between the sampling period and the period describing when the target species are active and therefore available for detection^10,15^. In our situation, this means aligning the sampling period of distance measurements with the period of time that desert bighorn sheep were active and able to trigger the trail camera^10^. In our study, we acquired very few nocturnal images of desert bighorn sheep (Fig. 4). Therefore, we censored study effort and distance data to the period defined by 1 h before sunrise through 2 h after sunset for each month, on a daily basis. Doing so removed < 2% of all images. This outcome matches the diurnal behavioral patterns of desert bighorn sheep^19^. Camera traps operating at night are also likely to have different detection functions than when operating during the day, potentially requiring separate model constructs for analyses.

We modeled detection functions using the key function (Uniform, Half-normal and Hazard-rate) + series expansion (cosine, simple polynomial, Hermite polynomial) approach^15^. These models provide estimates of detection probabilities, which are combined with encounter rate and survey effort to derive an abundance estimate^15^. Survey effort for camera trap-based surveys is the number of camera triggers possible per trap during the survey period (i.e., total number of seconds a camera was operating divided by trigger speed). We calculated abundances for desert bighorn sheep for each age and sex category and all adult categories combined. Competing models with sufficient goodness of fit (GOF) were selected using AIC criteria. Competing models (i.e., models within 2 ΔAIC) were model averaged. All analyses operated with α = 0.1.

Lastly, we performed an analysis to identify the number of sampling sites (i.e., camera traps) required to provide a coefficient of variation (CV) spanning 10–50%. We examined data from studies spanning 2 (spring period) and 4 months (fall period), respectively.

Our study received approval by NMDGF and the United States Fish and Wildlife Service prior to conducting the work. The authors confirm that all methods and experiments were performed in accordance with the relevant guidelines and regulations of these agencies.

## Supplementary information

Supplementary Table S1.

Supplementary Figure S1.

Supplementary Figure S2.

## Data Availability

All data used in our analyses are included as a supplementary file (Excel).
