# Supplementary figures and images for: Accurate population estimation of Caprinae using camera traps and distance sampling
Source: Sci Rep. 2020 Oct 20;10:17729. doi: 10.1038/s41598-020-73893-5 (PMC7576118; doi:10.1038/s41598-020-73893-5)

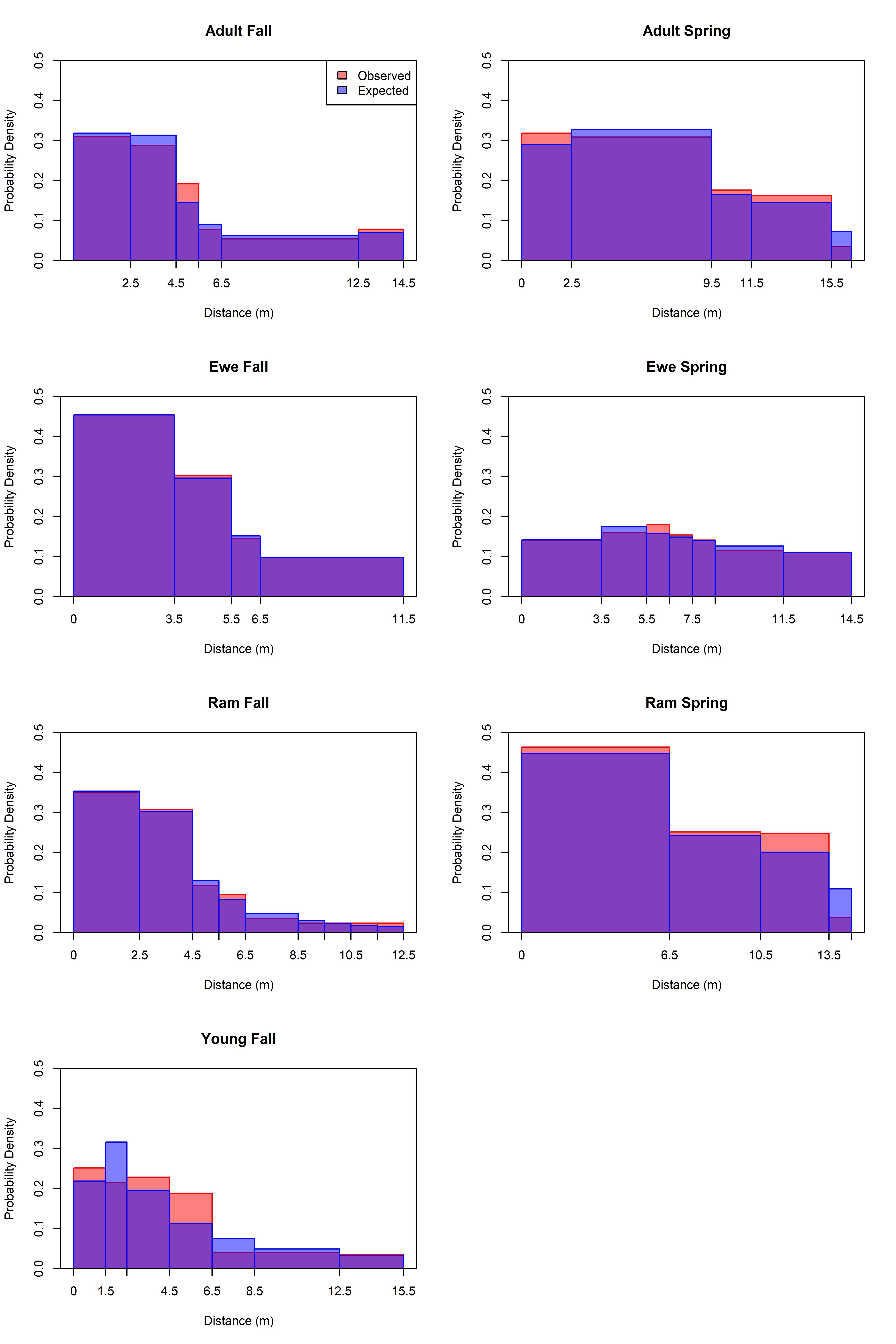

Supplement: Supplementary file 2 — Supplementary Figure S1. [file 41598_2020_73893_MOESM2_ESM.jpg]

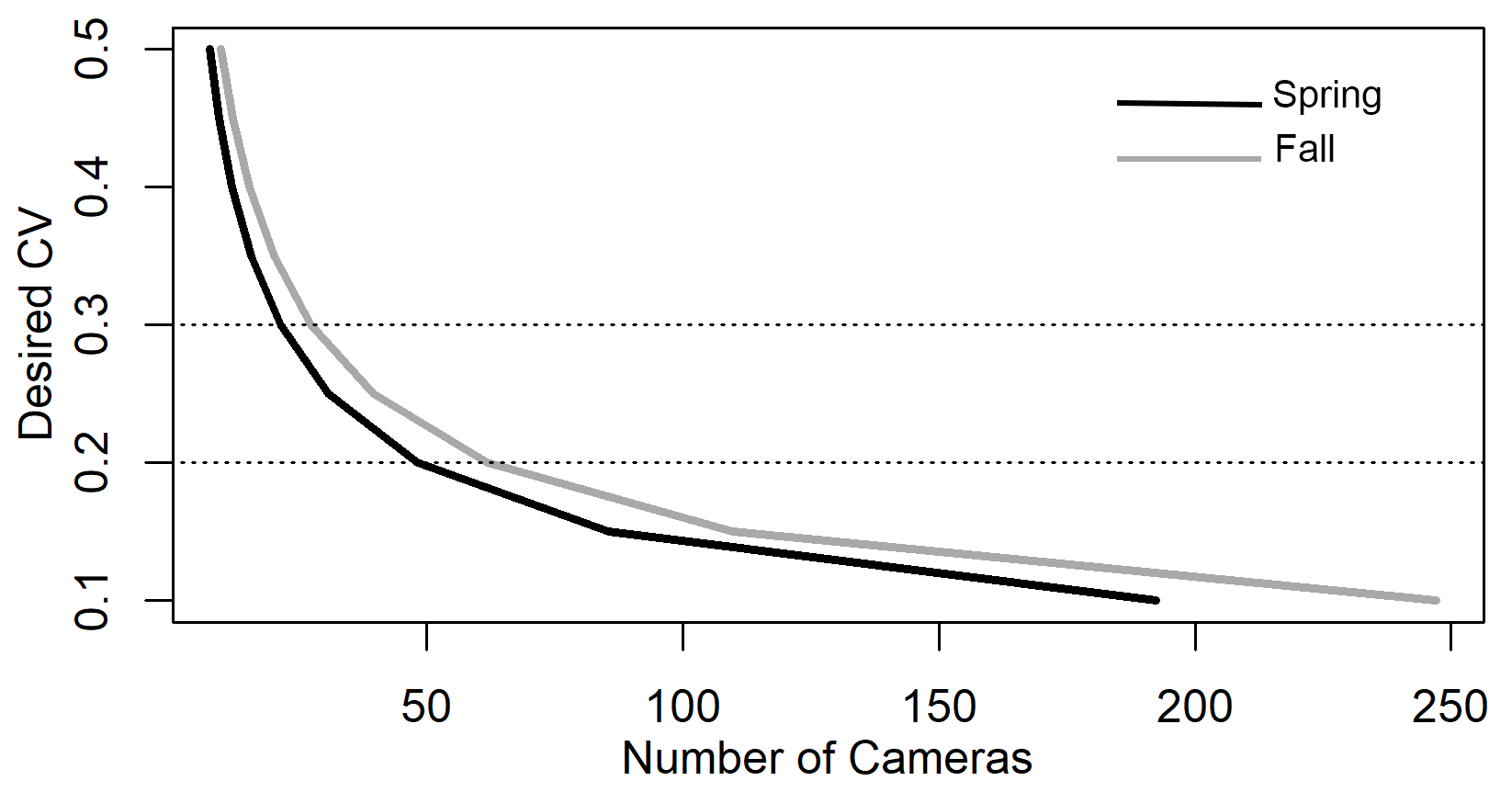

Supplement: Supplementary file 3 — Supplementary Figure S2. [file 41598_2020_73893_MOESM3_ESM.tif]
